# The Endothelial Centrosome: Specific Features and Functional Significance for Endothelial Cell Activity and Barrier Maintenance

**DOI:** 10.3390/ijms242015392

**Published:** 2023-10-20

**Authors:** Anton Sergeevich Shakhov, Aleksandra Sergeevna Churkina, Anatoly Alekseevich Kotlobay, Irina Borisovna Alieva

**Affiliations:** 1 A.N. Belozersky Institute of Physico-Chemical Biology, Lomonosov Moscow State University, 1–40, Leninskye Gory, 119992 Moscow, Russia; 2 Faculty of Bioengineering and Bioinformatics, Lomonosov Moscow State University, 1–73, Leninskye Gory, 119992 Moscow, Russia; 3 Lopukhin Federal Research and Clinical Center of Physical-Chemical Medicine of Federal Medical Biological Agency, 1a Malaya Pirogovskaya St., 119435 Moscow, Russia

**Keywords:** centrosome, centriole, endothelium, endothelial barrier function

## Abstract

This review summarizes information about the specific features that are characteristic of the centrosome and its relationship with the cell function of highly specialized cells, such as endotheliocytes. It is based on data from other researchers and our own long-term experience. The participation of the centrosome in the functional activity of these cells, including its involvement in the performance of the main barrier function of the endothelium, is discussed. According to modern concepts, the centrosome is a multifunctional complex and an integral element of a living cell; the functions of which are not limited only to the ability to polymerize microtubules. The location of the centrosome near the center of the interphase cell, the concentration of various regulatory proteins in it, the organization of the centrosome radial system of microtubules through which intracellular transport is carried out by motor proteins and the involvement of the centrosome in the process of the perception of the external signals and their transmission make this cellular structure a universal regulatory and distribution center, controlling the entire dynamic morphology of an animal cell. Drawing from modern data on the tissue-specific features of the centrosome’s structure, we discuss the direct involvement of the centrosome in the performance of functions by specialized cells.

## 1. Introduction

With the light hand of D. Wheatley, who titled his book “The Centriole: a central enigma of cell biology” four decades ago, the centrosome, which includes centrioles in its structure, has been recognized as, and to a large extent still remains, a mysterious structure [1]. There was a good reason for such a definition of the centrosome. By the time of publication, the main details of the chromosome structure and the principles of gene functioning had already been studied in detail. Nevertheless, the mechanism of the centrosome’s functioning, which surprisingly turned out to be a direct participant in many different processes in a living cell, remained mysterious. With the existing variety of functions, the centrosome’s structure practically does not differ in most living organisms. The centrosome’s functional universalism suggests a comparison of this cellular organelle with a computer processor, which, using the information that is stored on the “hard disk” in the nuclear chromosomes, organizes all the work of the cell.

This structure, which is now called the centrosome, was described for the first time in the seventies of the XIX century by several researchers of cell division at once [2,3,4]. It only looked like a dark granule in each of the poles of the mitotic spindle. The size of this organelle is almost at the resolution limit of a light microscope, at that time, it was not possible to study its detailed structure. Initially, two symmetrically arranged structures, having the form of a “radiant radiance”, and which were called centrospheres, were described in dividing cells. Granules, originally called polar corpuscles, were sometimes visible in the focus of each centrosphere [4]. It was found that polar corpuscles do not completely disappear at the end of mitosis but remain in the interphase, often located near the geometric center of the cell [5,6]. For this reason, E. Van Beneden and A. Newt proposed to rename them “central corpuscles” or “central bodies” [5], and T. Boveri proposed to call this structure a centrosome [6] and later a centriole [7].

It should be noted that the numerous names given by different authors to the same structure have created a terminological confusion. Before the era of electron microscopic studies, the terms “centriole” and “centrosome” were often used as synonyms. According to the modern terminology adopted in the literature since the mid-twentieth century, a centriole is a structure that is a part of a centrosome (a specialized zone of the cytoplasm located, as a rule, in the geometric center of the cell).

## 2. A Quick View on the Mammalian Centrosome Architecture

**Figure 1:**
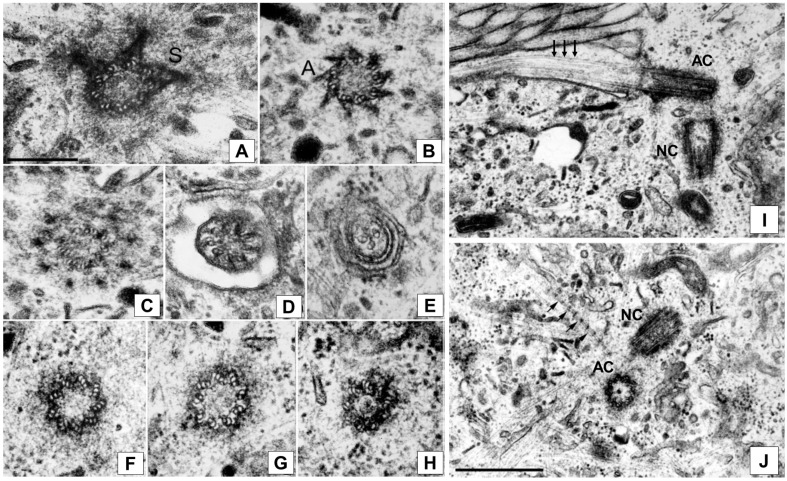
Electron micrograph of serial sections showing the fine structure of the centrosome and primary cilium (cross section) in PK (pig kidney) cell (G_0_ phase). Left panel: (**A**–**E**)—active centriole in the region of cilium appearance (sections 1, 3, 4, 5 and 8), S—pericentriolar satellites, A—appendages. (**F**–**H**)—non-active centriole (sections 1, 5 and 7). Bar = 0.2 μm. Right panel: fine structure of the centrosome and primary cilium (longitudinal section). The distal segment of the axoneme of primary cilium is out of the plane of section. (**I**)—active centriole (AC) (with the pericentriolar satellite, cilium (arrows)) and non-active centriole (NC). (**J**)—active (AC) and non-active (NC) centrioles with striated rootlet (arrows). Bar = 0.5 μm. Alieva, Vorobjev, 2004 [12] with modifications.

During the 1960–80s, when transmission electron microscopy became a routine method, the centrosome became a very popular research object. Electron microscopy studies have revealed the centrosome’s ultrastructure in general and its remarkable centriole architecture. The centrosome, a cellular organelle visible under a light microscope as a dense granule in the cytoplasm of a cell, possesses a complex ultrastructure that is common in most animal cells that have been studied. It is composed of a pair of centrioles (cylindrical structures consisting of nine microtubule triplets) surrounded by a pericentriolar material. It is worth noting that the centriolar cylinder microtubule triplets are very stable and, unlike cytoplasmic microtubules, they are not disassembled under the action of mitostatic agents (or antimitotic agents—cell division blocking drugs) or under the influence of cold [8,9,10]. Disassembly only occurs in isolated centrioles at high salt concentrations [11]. The centriole keeps its form after the triplet’s removal. The structure that appeared after such treatment was named the centriolar rim [11]. This assumes that the centriolar matrix, rather than microtubules, comprises the basis of centriolar cylinders. The centrioles of the pair are different: one of them is mature or maternal, and, unlike the second one, which is immature or the daughter, it bears additional structures, such as pericentriolar satellites and appendages (Figure 1) [12]. Procentriole assembly begins with the assembly of a nine-fold symmetrical structure, called a cartwheel, which helps establish procentriole symmetry. The cartwheel is, in vertebrate cells, removed from the procentrioles in mitosis [13]. Another difference of the maternal centriole is its ability to convert to a basal body and form a primary cilium, which is a sensory organelle, which is often associated with striated rootlets. The older centriole can, usually after exiting the cell cycle, convert to a basal body and form a primary cilium. Primary cilia are not present in all cell types. Their function is to transmit signals between the extracellular environment to the cell interior, and they are critically important for tissue homeostasis and development [14,15,16,17]. The primary cilium is critically important for specialized cell functions. Motile cilia move sperm [18,19]. In differentiated epithelial multiciliated cells, primary cilia promote the flow of fluids that are critical for mucus clearance, promote left–right patterning during development, or transport the egg cell from the ovary to the uterus [15,20,21,22]. The mature centriolar cylinder is about 0.3–0.5 µm in length and 0.2 µm in diameter. Together with the abovementioned components, the centrosomes of some cell types may contain additional structures, such as free foci of microtubule convergence. The elements comprising the centrosome themselves possess a complex constitution. Detailed electron microscopy analyses of the morphological aspects of the centrosome structure have been presented in numerous investigations, and, although the centrosome may have specific features in a number of differentiated cells and can undergo remodeling and reduction in germ line cells, it has been generally accepted that, with the exception of some details, the structure of the centrosome is quite uniform [8,11,13,23,24,25,26,27,28,29,30].

Biochemical and immune-cytochemical studies have shown that the centrosome is a multiprotein complex. In addition to tubulin, which is part of the microtubule triplets, about 600 proteins have already been found in the centrosome [31]. Part of the centrosomal proteins can be classified as the structural proteins either directly involved in the centriolar structure or localized in the pericentriolar matrix. Another centrosomal protein may be either permanently associated with the centrosome or appear in its structure in the distinct cell cycle stages. In addition, the centrosomal proteins can be classified by their functions: protein motors, cell cycle regulatory proteins, components of microtubule nucleation complex, etc.

Modern proteomics and genomic screens conducted in multiple species identified hundreds of centriole and centrosome core proteins and revealed the evolutionarily conserved nature of the centriole assembly pathway in numerous species. Contemporary super-resolution microscopy approaches [32,33,34] and improvements in cryo-electron tomography are bringing an unparalleled picture of the centriole and centrosome architecture nanoscale detailed [35,36,37,38,39,40].

As the name (the centrosome—‘central body’) suggests, the default position of the centrosome is considered to be the cell geometrical center. However, the mechanism regulating centrosome positioning is still unclear. It has been shown that acto-myosin network geometry defines centrosome position and microtubules play an important role in centrosome positioning. It was shown that in the epithelium the centrosome is kept at the center by a pulling force generated by dynein acting along microtubules and actin flow produced by myosin contraction [41,42]. The mechanism of centrosome movement in the endotheliocyte is not fully understood today but apparently it is also associated with motor proteins, as well as some linker proteins, and small GTF-ases (Cdc42, RhoA, Rac1, etc.) can act as key regulators.

## 3. Constancy of Architecture and Tissue-Specific Functioning Features: Can Multiple Centrosome Activities Be Selectively Modulated to Perform Specific Cellular Functions?

Despite the universality of the centrosome structure in mammalian cells (with extremely rare exceptions, like sperm cells) [43], it always includes a pair of nine-fold symmetrical microtubule-based centrioles which organize the pericentriolar material [13,44,45,46,47,48]. The literature accumulates data indicating that specific features in the centrosome organization (the presence/absence of centriole-associated structures, such as distal appendages and subdistal appendages (pericentriolar satellites), primary cilium etc.) and its activities are observed in cells of various tissues, which is characteristic only for this cell type [28,29,49]. And, although the structural or genetic reasons (like specific genes overexpression) causing such features are still unknown, the authors reasonably associate these features with the functions performed by the centrosome in this cell.

In recent years, studies have appeared demonstrating the direct involvement of the centrosome in the processes of cellular differentiation and morphogenesis [50]. At the same time, either activation or inactivation of the centrosome occurs at different stages of the processes.

At the beginning of neuronal morphogenesis, before polarization, microtubules emanate through the soma from the active centrosome. In the early stages of neuronal polarization, the axon is specified from the branch closest to the active centrosome but at later stages, the centrosome is inactive [51]. The centrosome’s position in the cell varies during neuron development. During proliferation, it is attached next to the nucleus. In newly polarized neurons, the centrosome is an active microtubule-organizing center with a dynamic position in the cell, but over time, it gradually loses its activity. In mature neurons, the microtubule cytoskeleton is not linked with the centrosome [52]. During dendritic arborization, the centrosome has been shown to be important as a nucleator of other factors/complexes necessary for dendrite formation [53].

Experiments performed on mammalian cortical neural precursors demonstrate the importance of centrosomal activity for the development of neurons and the maintenance of cortical neurogenesis [54,55]. Likewise, the centrosome is an important player in single-cell branching morphogenesis. Centrosomes are active microtubule-organizing centers enabling the correct microtubule system organization necessary to start forming the extending subcellular lumen. Centrosome activity is critical during the first steps of subcellular lumen formation. But later, when the lumen is already formed, centrosomes become inactive and non-centrosomal microtubule polymerization sites are switched on [51].

Thus, the activity of the centrosome can be selectively modulated in cells under the influence of factors which are already known. To a subject of our interest—the endothelium, then a number of angiogenesis inducers are known [56]. Some factors stimulating angiogenesis do this indirectly by binding to the protein β3-endonexin which is associated with the centrosome. This protein interacts and co-localizes with ninein, a proangiogenic centrosome protein located in human umbilical vein endothelial cells [57,58,59]. Interacting with centrosome ninein, β3-endonexin contributes to the proliferation of vein endothelial cells and the formation of vascular structures.

The main function of endothelial cells closely bordering one another and spreading on the inner vessel surface is the regulation of vascular tonus and vascular wall permeability, which ensures the exchange between the blood circulating inside the vessels’ and organs’ tissue fluid. Thus, the eye corneal endothelium is responsible for the normal permeability and exchange of the retina with the connective tissue stroma [60], whereas the pulmonary vessels’ endothelium regulates the fluids’, macromolecules’ and leukocytes’ movement into the interstice and alveoli aerial space [61,62,63].

Since the main function of endothelial cells is to provide a semi-permeable barrier between the blood circulating in the vessel and the tissues adjacent to the vessel, an obvious question arises: are there specific features in the organization of the endotheliocyte centrosome that can potentially be involved in maintaining specific endothelial functions, including the barrier? A number of studies indicate that the native structure of the endothelial centrosome is essential for the normal function of endotheliocytes [64]. Factors that regulate both angiogenesis and endotheliocyte centrosomal activity have been described. Endothelial cells that were exposed to levels of FGF and angiogenic factors expressed excess centrosomes, indicating that multiple factors are involved in the regulation and doubling of centrosome numbers [65].

A number of structural features of the endothelial centrosome make it the main active and responsive microtubule organization center, being directly involved in the barrier function performance. Earlier, we expressed the idea that the endotheliocyte centrosome, due to its architecture peculiarities, has the ability to quickly activate, thus serving as a center for the integration of signaling pathways, as well as coordinating all cytoskeletal endothelial systems [66]. Below, we discuss the structured organization of the endothelial centrosome during the interphase, focusing on the localization/function relationship.

## 4. Ultrastructural Features of Endothelial Cell Centrosome

The structure of the centrosome in various endothelial cells began to be actively studied in the 80s of the XX century. Thus, ultrastructural features of human vascular endotheliocytes centriolar complexes in situ were studied in detail in connection with various cardiac pathologies in patients aged 50–60 years [67]. The authors described the structure of “healthy” endotheliocyte centrosomes located in intact zones, as well as in fibrous and atheromatous areas of the artery from human autopsy material in situ.

It was discovered that five types of centrosomes with different structural features can be distinguished [67]. In one of the selected groups, the structure of the centrioles themselves changed: no cartwheel-type structures were found inside the centrioles and one or two microtubules in the triplet were missing. However, in all types of cells, the maternal centriole carried a significant number (from 2 to 12 per centriole) of satellites, as well as distal appendages. The activity of centrosomes in relation to primary cilium formation also differed. Most endotheliocytes from atheromatous regions had a primary cilium, while in fibrous and intact zones such cells were rare. The authors suggested that the observed difference in the centrosome’s structure may be due to functional differences [67].

Studies have shown that in the endothelial cells of the human aorta taken during autopsy in younger patients (14–17 years old), the centrioles that make up one centrosome were variable in length, and in the distal part of the maternal centriole, doublets could be observed instead of microtubule triplets [68]. In the aortic cells of donors who were 30–40 years old, there were centrioles with completely defective walls, consisting of microtubule doublets for the entire length [68]. In endotheliocytes from embryonic material (age 22–24 weeks), the length of the maternal and daughter centriole cylinders was the same (0.5–0.6 microns) [68].

One of the results of the studies described above is the fact that the activity of the endotheliocyte maternal centriole in situ may vary depending on the condition of the vessel. In particular, the number of pericentriolar satellites responsible for the polymerization of microtubules diverging from the centrosome varies significantly. Since ethical standards and the limited human biomaterial availability significantly hindered the research, most of the experiments were carried out on the endotheliocyte culture. This model, which ensures the experimental conditions’ stability and their reproducibility, allowed researchers to make significant progress in understanding how important the centrosome is in ensuring the vital endothelial cell activity.

In endothelial cells in vitro, most of the endotheliocyte microtubules are anchored on the centrosome, which organizes them into a radial system [66,69,70]. The minus ends of the microtubules can be fixed on the head of the subdistal appendage (satellite) or can be located in the pericentriolar material, being capped by the γ-TuRC complex. The plus ends of microtubules progressively grow from the centrosome towards the cell edge, and their growth rate significantly exceeds the growth rate of the plus ends of a few free microtubules.

Studies conducted in vitro on isolated cells of the human aorta and vein indicate that the endotheliocyte centrosome can quickly (on a minute scale) respond to various chemical influences, responding with pronounced biochemical and even morphological changes. It was discovered that the ratio of acetylated and tyrosinylated α-tubulin in the composition of centriolar cylinders can change in response to stimulation by thrombin, which is capable of inducing endothelium barrier dysfunction [71]. The speed with which the response develops is remarkable. Already after 1 min of exposure, a significant increase in the intensity of centrioles staining on acetylated tubulin was observed, and the intensity of staining on tyrosylated tubulin significantly decreased 5 min after exposure [71]. An ultrastructural analysis of human artery endotheliocytes showed that the centrosome morphology changes just as quickly in response to thrombin exposure, and additional pericentriolar satellites are formed on the maternal centriole. After 3 min of thrombin exposure, the number of satellites increased to 5–6 (normally from 1 to 4), and no centrioles with less than 3 satellites were observed. The described effect persisted after 15 min of exposure. Paradoxically, when thrombin was exposed to human vein endotheliocytes, no additional satellites were formed on the maternal centriole, and their number remained close to the control values [71].

**Figure 2:**
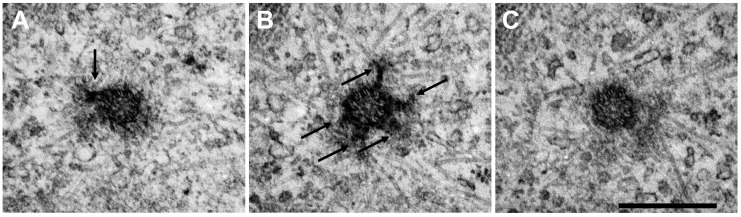
The ultrastructure of active (maternal) centriole in the centrosome of endothelial cell on cross sections ((**A**–**C**)—sections 2, 3, 4.) Endothelial cell centrosome is a very active microtubule-nucleating center. Maternal centriole (shown) has 6 satellites (indicated by arrows). Numerous microtubules grow away from both heads of pericentriolar satellite and centriole walls. Human pulmonary artery endothelial cell (HPAEC). Electron microscopy. Bar 0.5 µm. Shakhov et al., 2017 [72] with modifications.

An electron microscopy analysis of a human pulmonary artery endothelial cell undertaken by us showed us that centrosomes are very active microtubule-nucleating center: a lot of satellites are located on the maternal centriole and numerous microtubules grow away from both heads of the pericentriolar satellite and centriole walls (Figure 2) [72].

Thus, the endotheliocyte centrosome is characterized by the presence of a larger (compared to other cells) number of pericentriolar satellites—structures that provide the active polymerization of microtubules. The endotheliocyte centrosome can respond quickly to external influences that lead to a violation of the barrier function. It stabilizes the structure of centriolar cylinders and increases the number of structures that are responsible for the polymerization of microtubules. It can be assumed that the described changes are a compensatory reaction in response to the depolymerization of microtubules, which is characteristic of the barrier dysfunction caused by thrombin [73].

## 5. Position and Intracellular Movement of Endothelial Centrosome

The experimental results presented above demonstrate that the centrosome’s position varies during proliferation and morphogenesis [52,53] Thus, for normal participation in these processes, the centrosome must actively move in the cell volume. Interestingly, the mobility of the endotheliocyte centrosome became one of the first questions concerning its properties and behavior in the cell and was actively investigated in the eighties of the last century.

Studies related to the position of the centrosome in the endothelial cell were especially actively conducted in in vitro experiments on a model of an experimental wound of aortic endotheliocyte monolayers [74]. The results suggested that the centrosome may play an important role in the directional movement of endothelial cells. After applying the experimental wound, the centrosomes in the cells changed their random position as follows: in 80% of endotheliocytes, the centrosome shifted to the area in front of the cell nucleus in the leading-edge area. This reorientation of the centrosomes coincided with the moment of active endotheliocytes moving into the wound [74]. A similar reorientation of the centrosome occurs when exposed to cytochalazine B, which damages the actin cytoskeleton of the cell; although, in this case, the centrosomes move more slowly, and the cells lose their movement direction [75]. However, when the microtubules were destroyed, centrosome reorientation was not observed. The displacement of the centrosome towards the leading edge was possible only under conditions when an intact microtubule system was preserved [75].

The experiments conducted on rabbit and pig vessels in vivo, remarkable in the complexity and originality of the design, obtained very interesting results, illustrating the critical importance of the centrosome’s specific position in the vessel endotheliocyte and its active movement to restore this position [76]. Normally, in most endotheliocytes (≈80%) of rabbit and pig inferior vena cava, centrosomes are located mainly in front of the nucleus: as the authors point out—in the direction of the heart, i.e., in the blood flow direction. To determine whether this centrosome’s position is preserved, the authors performed a microsurgical operation: they cut out a segment of the rabbit’s inferior vena cava, turned it in the opposite direction and implanted it back. It was found that after the reversal in the implanted fragment, there was a gradual restoration of the original centrosomes’ orientation. A week after the operation, the centrosomes moved to a more “central” position, and after 12 weeks, the centrosomes’ position corresponded to normal—they were located in front of the nucleus [76]. The above experimental data confirm that the centrosome plays a special role in the endotheliocyte and for normal endothelium functioning in vessels in vivo, the cell must maintain a strictly defined centrosome position.

A convenient model system for the endothelial monolayer study turned out to be a pig’s thoracic aorta organ culture. Experiments carried out on this model system confirmed the centrosome’s participation in the endothelial cells’ movement. If the endothelium was not exposed, then the centrosome in the endotheliocytes gradually moved from the position “to the heart” to the cell center. In the conditions of the experimental wound, the centrosome shifted towards the leading edge [77,78]. The centrosome displacement, although slower, also occurred if a cell culture was used instead of an organ one [78]. The centrosome’s movement to the leading edge of the endotheliocyte coincides in time with the beginning of cell movement towards the experimental wound.

**Figure 3:**
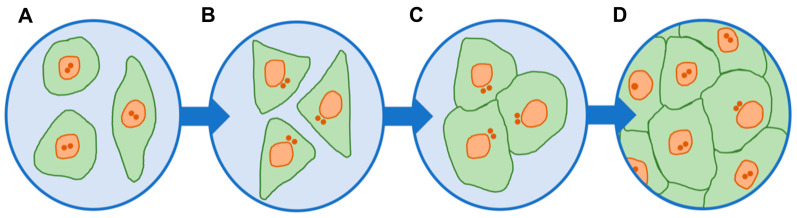
Endothelial cell centrosomes change their position in endothelial monolayer formation in vitro. (**A**) centrosomes are located in the geometric center of the at rounded cells (stage of spreading). (**B**) centrosomes are located predominantly in front of the nucleus (stage of polarization). (**C**) centrosomes are displaced towards the forming VE-cadherin contact (inter endotheliocytes contact less than 70%). (**D**) centrosomes return to the central position (inter endotheliocytes contact more than 70%).

Our own studies conducted on HPAEC and EA.hy926 (a hybrid line derived from human endothelium and A549 cells) cell lines in vitro showed that at the spreading stage, when the cells are rounded, the centrosome is located in the geometric center of the cell (Figure 3). When cells are polarized under conditions of rare seeding cell culture, when cells are located at a great distance and do not have contact with each other, the centrosome shifts to the leading edge of the cell, and regardless of the shape acquired during the transition to movement (fibroblast-like or keratinocyte-like), it is located predominantly in front of or above the nucleus. The shift of the centrosome is observed at all stages of the formation of the endothelial monolayer, up to the final one; however, when the VE-cadherin contacts between adjacent endotheliocytes are formed by more than 70%, the centrosome returns to the central position (Figure 3). Thus, the centrosome’s movement to the leading edge of the endotheliocyte coincides in time with the beginning of cell movement not only during directional movement (experimental wound), but also during the random wandering of endotheliocytes in the early stages of monolayer formation, giving grounds to consider the centrosome as an important link in the initiation of endotheliocyte movement.

The reorientation of the centrosome to the region between the nucleus and the cell free edge is an accompanying event for cellular polarization and subsequent endotheliocyte migration. The change in the centrosome position in the regenerating monolayer in vitro can be blocked by bFGF inhibitors. Suppression of the centrosome reorientation process is accompanied by a decrease in the number of stress fibrils in the endotheliocyte cytoplasm [79]. If microtubule disassembly is artificially blocked, the ability of a cell to migrate is suppressed, with the observed number of cells with centrosomes shifted towards the free edge being significantly reduced [80]. Later, it was shown that not only microtubules but also the endotheliocyte actomyosin system are involved in the centrosome moving process. At the same time, their mutual regulation is possible: in endotheliocytes with defective centrosomes, the activity of the small GTF-ase Rac1 increases, but the effect of nocodazole, which destroys microtubules and prevents their nucleation, eliminates this effect. The blocking of Rac1 and/or Arp2/3 leads to a more ordered centrosome orientation in defective cells [81].

In addition to the fact that the centrosome’s intracellular movement is regulated by the small GTPase Rac1, other regulatory proteins are also known. Regulation of the endotheliocyte’s polarization and migration can be carried out with the help of the HOP protein (Hsp70/Hsp90-organizing protein), which is capable of binding to the chaperones Hsp70 and Hsp90 and modulating their ATPase activity. Under HOP knockdown conditions, the dynamics of the endotheliocyte active edge decrease, but the expression of the Hsp90 partner proteins (Cdc42, RhoA, Rac1, Akt and HDAC6) that are responsible for endotheliocyte dynamics does not change [82]. HOP knockdown suppresses the formation of tube-like structures in the 2D culture of HUVEC cells cultured on matrigel, while HOP overexpression enhances their growth. If HUVEC cells are cultured in 3D culture, partially suppressing HOP, capillary-like structures are formed but are much shorter than in the control [82]. HOP also plays an important role for endothelial cell movement; its knockdown significantly weakens the ability of endotheliocytes to migrate towards the wound. Conversely, overexpression stimulates this process. In HOP-deficient cells, the centrosome was located randomly, while normally the centrosome shifted towards the endotheliocyte active edge. At the same time, HOP is able to bind to tubulin and microtubules, which may indicate its possible role in the regulation of some centrosome activities [82].

The above data became the basis of the idea that the centrosome is an arrow of some kind of intracellular compass, of which the shift indicates the direction of the cell movement. There is also an alternative point of view, according to which centrosome loss had no effect on the ability of endothelial cells to polarize and move [83]. According to the results obtained by these authors, centrosomes did not affect cell migration, but getting rid of CAMSAP2 did. Analysis of cell shape and movement in cells grown in the laboratory and in living animals revealed that cells cannot become asymmetrical, or “polarize”, and migrate without CAMSAP2. The authors believe that the key regulators of the process are non-centrosomal microtubules stabilized by the microtubule minus-end binding protein CAMSAP2 were vitally required for directional migration on 2D substrates and for the establishment of polarized cell morphology in soft 3D matrices.

## 6. Endothelial Centrosomes: Over-Duplication in Tumor Vessels and in Angiogenesis

Centrosome abnormalities are a general property of many cancer cell types. It is known that tumor progression requires angiogenesis, which is a hallmark of cancer development, and tumor vessels enable tumor metastasis by providing a conduit for tumor cell invasion and spread [84,85]. Endothelial cells that line tumor vessels have genetic abnormalities, such as aneuploidy, which is often associated with excess centrosomes, and approximately 30% of tumor endothelial cells have over-duplicated (>2) centrosomes, which may contribute to abnormal vessel function and drug resistance [64,86,87]. It was shown that some tumor-derived factors and genetic changes in endothelial cells contribute to excess centrosomes in tumor endothelial cells which may contribute to abnormal vessel function and drug resistance [88].

Excessive centrosome duplication can be observed not only in vascular tumor cells but also amongst endotheliocytes of a growing vessel. The VEGF-related signaling pathway plays an important role in this process. The loss of the VEGFR-1 receptor gene (Flt-1) entails disordered hypervascularization and a decrease in vascular branching, and in mice mutant embryonic cells deficient in the Flt-1 receptor (flt-1-/-), it significantly increases the number of endothelial cells with an excessive centrosome number [65,89]. HUVEC cells containing excess centrosomes survived and divided mitotically, generating an accumulation of cells with excess centrosomes which disrupted the normal vessel functioning [65].

At the moment, it is not completely clear how the excessive centrosome number in interphase cells affects the activity of the centrosome in relation to the microtubule system organization during the interphase and mitosis, as well as the microtubules’ dynamics and cellular mobility. It was discovered that endothelial cells (with an excessive centrosome number) not only had reduced mobility but also lost the ability to reorient. As a consequence, the ability to form new vessels significantly decreased in HUVEC cells cultured in 2D or in 3D after induction of the additional centrosomes’ formation using Plk1 overexpression [90].

Centrosomal proteins involved in angiogenesis are described [59,66,91]. An important regulator of normal angiogenesis is the centrosomal protein Cep70. The knockdown of Cep70 led to a significant angiogenesis suppression in HUVEC cell culture and reduced the endotheliocytes’ ability to migrate (both when crawling into the experimental wound and during the capillary-like structure’s formation); at the same time, it also critically affected the centrosome location, which moved from the typical position in front of the endotheliocyte nucleus to the position behind the nucleus [92]. These researchers suggested that the role of Cep70 as a cell polarization regulator is realized using Rho-GTPase Rac1 and Cdc42 but not RhoA, i.e., Cep70 can act as an Rac1 and Cdc42 activator [92]. Another centrosomal protein involved in angiogenesis may be the FAK kinase, which is part of the centrosome during mitosis and regulates focal contacts [91].

Experiments with the HUVEC endothelial monolayer on the effects of lonafarnib, which is a potential neovascularization inhibitor in atherosclerotic plaques, perfectly illustrate the centrosome’s involvement in the endothelial cell polarization process and angiogenesis [93]. Lonafarnib is capable of (1) interrupting the capillary-like structure’s growth on the matrigel; (2) causing a dose-dependent decrease in endotheliocyte motility; and (3) disrupting the centrosome reorientation (in polarized endotheliocytes, the centrosome is located randomly and not between the nucleus and the cell leading edge) [93].

The plant alkaloid EM011 is apparently another chemical agent affecting angiogenesis and endotheliocyte polarization. It is able to bind to tubulin and simulate the microtubules’ dynamics and to delay mitosis without disturbing important physiological functions in which microtubules are involved [94]. EM011 disrupts the tube-like structure’s formation in HUVEC cell culture and inhibits the vessel’s formation and development in vivo in mice and fishes. EM011 changes the centrosome’s position in migrating HUVEC cells to random, hinders the filopodia, lamellipodia and stress fibrils formation, and reduces the RhoA, Rac1 and Cdc42 expression [94].

Thus, the centrosome native state in the endotheliocyte is essential for normal angiogenesis, and endothelial centrosome over-duplication leads to a whole spectrum of disorders.

## 7. Structural Disorders of Centrosomes in Endotheliocytes Leading to Endothelial Barrier Dysfunction

Based on our own long-term experience and other researcher’s data, in this review, we have presented a large number of studies confirming the involvement of the centrosome in the functioning of endotheliocytes and emphasizing the critical importance of maintaining its native structure and the ability to quickly activate to perform these functions.

Changes in the centrosome’s structure can disrupt not only the cellular differentiation and morphogenesis processes, and angiogenesis, but also provoke centrosomes’ involvement in the chain of reactions that ensure the endothelial barrier integrity [71]. The centrosome changes structurally in response to the factors causing endothelial dysfunction and these changes occur quite quickly, within a few minutes. The induction of excess centrosomes by overexpression of Plk1 or Cdc14B knockout in HUVEC cells causes not only a decrease in endotheliocyte motility when crawling into an experimental wound. The presence of cells with amplified centrosomes directly affects the basic endotheliocytes’ barrier function, increasing the endothelial monolayer permeability [64]. Amplified centrosomes had defects in the pericentriolar material, which led to a decrease in the number of polymerizing microtubules. Their growth was disordered, as a result of which the microtubule system polarization as a whole was disrupted [95].

Another study of endotheliocytes with excess centrosomes revealed abnormal microtubule dynamics: there were significantly more catastrophes and fewer microtubules in a stable state than in the control [81]. The microtubule system of endotheliocytes with excessive centrosomes responds in an original way to cold exposure. At the beginning of exposure, the density of microtubules increases and then decreases significantly compared to the control, which may indicate the involvement of a new signaling pathway in the reaction [81]. The experimental decrease in the microtubule number in the VE-cadherin contact area, which we described earlier, as well as a decrease in the centrosome activity with respect to the microtubules’ polymerization, leads to a violation of the endothelium barrier function [73,96,97].

The above experimental data confirm that the normal structure of the endotheliocyte cell center is essential for the endothelium’s functioning. An excessive centrosome number in interphase cells leads to a change in the entire microtubule system dynamics and (indirectly) affects cellular mobility, as well as the barrier function maintenance by the endothelium.

## 8. The Centrosome of Endotheliocytes, Being an Active Center of Polymerization of Microtubules, Coordinates the Architecture of the Cytoskeleton through Dynamic Microtubules

Traditionally, the main function of the centrosome at different cell life stages has been, and continues to be, considered its ability to nucleate and organize the cytoplasmic microtubule’s system and play a decisive role in intracellular transport, cellular mobility and mitosis [95,98]. Endothelial cells are a classic example of the microtubule’s system organization. The centrosome in these cells is the main center of the microtubule’s polymerization and organization [70,73]. In the cells of the endotheliocyte monolayer, the centrosome is located in the center, providing the organization of the radial microtubule system. Such organization of the microtubule system is essential for performing endotheliocytes’ barrier function: (1) the structure of the endotheliocyte centrosome ensures its high activity and ability to respond to external influences; (2) a radially organized microtubule system ensures the uniform presence of microtubule plus ends on the cell periphery in the area of intercellular contacts; (3) anchoring the microtubule’s negative ends on the centrosome protects them from disassembly and allows the plus ends to grow progressively to the cell edge and regulate VE-cadherin contact.

Our knowledge of the centrosome is supplemented by information about its relationship with other elements of the cell cytoskeleton. Actin [99] and the CapZ protein copying actin [100] were found in the interphase centrosome, and the temporary presence of the Arp2/3 complex in the pericentriolar material was also recorded [99]. In addition, cortactin, motor and other proteins that are direct regulators of actin in the cell were found in the centrosome composition [101,102,103]. Recent studies conducted on centrosomes in vitro suggest that, under certain conditions, the centrosome can function as an organizer of actin filaments [101]. The interaction of different cytoskeletal systems with the centrosome can be carried out by means of cross-linker proteins and signaling cascades, including known regulators of the cell cytoskeletal systems. One of the actin isoforms, γ-actin, can affect the centrosome’s position. In γ-actin-deficient neuroblastoma cells, the normal reorientation of the microtubule organization center is disrupted under experimental wound conditions [104]. Partial depletion of γ-actin can cause excessive centrosome amplification in cancer cells and delay the mitosis passage [105]. It is obvious that the experimental data obtained in recent years on the centrosome’s interaction with the cell γ-actin structures and on the signaling cascades associated with this interaction are universal for cells of various types, including endothelial cells [106]. As we now understand, all cytoskeletal systems are directly or indirectly involved in the implementation of the main function of endothelial cells, and the existence of cross-linker proteins suggests mutual regulation of cytoskeletal components. The actin system and microtubules are the most important links involved in maintaining the endothelial barrier. In vitro studies of the endothelium have demonstrated the special role of the actin and microtubules’ interaction for the repair and maintenance of the endothelial monolayer. Moreover, microtubules were also necessary to maintain a properly organized microfilaments network [107]. It was found that under the influence of factors causing a barrier function violation, first the depolymerization of microtubules on the endotheliocyte edge occurs, and then actin stress fibrils appear in the cytoplasm [73,96,97].

There is an assumption that the centrosome can serve as the center of the intermediate filaments’ organization and be responsible for their distribution in the cytoplasm [108,109]. In recent years, it has been shown that vimentin filaments are able to bind to both microtubules and actin filaments [110], and the protein plectin can be involved in the interaction of vimentin filaments and centrosomes [82,111]. The connection of vimentin and actin filaments, carried out with the plectin help, is extremely important for cellular morphogenesis [112], and plectin is able to interact with the BRCA1 protein and form a complex that controls the centrosome position [112]. It is possible that the vimentin network and plectin may participate in the positioning of the centrosome in endothelial cells [65]. According to the proposed model [65,66], there is a vimentin filaments network around the centrosome, connected to each other with the help of plectin and localized near the cell nucleus with the help of the nesprin-3 ligament protein. Apparently, plectin contributes to maintaining the endothelial monolayer’s integrity; it can regulate the interaction of the vimentin network and actin filaments [113]. When culturing plectin-deficient cells, large gaps in the endothelial monolayer were observed, which could probably be due to a violation of the actin filament’s connection with VE-cadherin contact [113].

**Figure 4:**
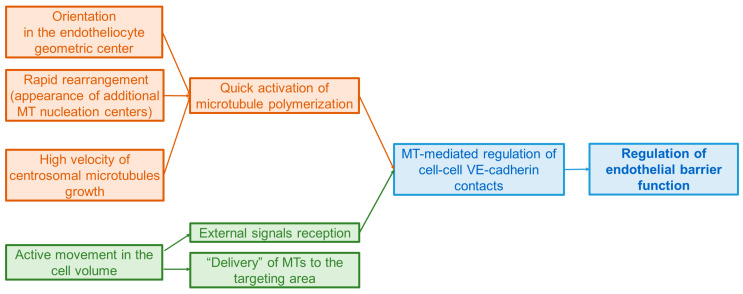
Structural and behavioral features of the endothelial cell centrosome allow it to react quickly (tan), on a minute scale, to external influences (green), responding with morphological changes and providing the barrier function regulation (blue).

Summarizing, the endothelial cell centrosome plays an essential role in endothelial barrier maintenance not only because of its centrality in the microtubule nucleation and radial organization, but also due to its important integrating function (Figure 4). By the mediation of the primary cilia, it is able to accumulate external signals and regulate vesicular transport to the membrane: on the one hand due to the rapid increase in the number of pericentriolar satellites regulating the density cytoplasmic microtubules through stimulation of their polymerization, and on the other hand, promoting the secretion of proteins involved in the formation of VE-cadherin contact. The centrosome serves as a center for the integration of endotheliocyte signaling pathways; it coordinates all cytoskeletal systems of endothelial cells involved in this process.

## 9. Conclusions

The special set of ultrastructural features of the endothelial cell centrosome allows it to react quickly (on a minute scale) to external influences, responding with pronounced biochemical and morphological changes. The determinacy of the centrosome’s position in the endotheliocyte in some functional states, and the ability to actively move in the cell volume to perform other functions, allows us to consider the centrosome as part of the intracellular mechanism responsible for morpho-functional reactions of the cell that go beyond the function of the microtubule’s organizing center. The endotheliocyte centrosome is not only the main active and rapidly reacting center for the organization of microtubules that is directly involved in the barrier function performance, but also serves as a center for the integration of endotheliocyte signaling pathways. The centrosome coordinates all cytoskeletal systems of endothelial cells involved in this process to varying degrees.

## Data Availability

The data presented in this study are available in https://pubmed.ncbi.nlm.nih.gov/.
